# Evaluation of the Validity and Reliability of Connected Insoles to Measure Gait Parameters in Healthy Adults

**DOI:** 10.3390/s21196543

**Published:** 2021-09-30

**Authors:** Damien Jacobs, Leila Farid, Sabine Ferré, Kilian Herraez, Jean-Michel Gracies, Emilie Hutin

**Affiliations:** 1 FeetMe S.A.S., 157 bd. MacDonald, 75019 Paris, France; leila.farid@feetme.fr (L.F.); sabine.ferre@feetme.fr (S.F.); 2 UFR de Mathématiques, Université Pierre et Marie Curie, 75005 Paris, France; kilian.herraez@gmail.com; 3 Laboratoire Analyse et Restauration du Mouvement (ARM), Hôpitaux Universitaires Henri Mondor, Assistance Publique des Hôpitaux de Paris (AP-HP), 94000 Créteil, France; jean-michel.gracies@aphp.fr (J.-M.G.); emilie.hutin@aphp.fr (E.H.); 4 EA 7377 Bioingénierie, Tissus et Neuroplasticité (BIOTN), Université Paris-Est Créteil (UPEC), 94000 Créteil, France

**Keywords:** validation, insoles, GAITRite^®^, pressure sensor, IMU, gait, gait variability, FeetMe^®^

## Abstract

The continuous, accurate and reliable estimation of gait parameters as a measure of mobility is essential to assess the loss of functional capacity related to the progression of disease. Connected insoles are suitable wearable devices which allow precise, continuous, remote and passive gait assessment. The data of 25 healthy volunteers aged 20 to 77 years were analysed in the study to validate gait parameters (stride length, velocity, stance, swing, step and single support durations and cadence) measured by FeetMe^®^ insoles against the GAITRite^®^ mat reference. The mean values and the values of variability were calculated per subject for GAITRite^®^ and insoles. A *t*-test and Levene’s test were used to compare the gait parameters for means and variances, respectively, obtained for both devices. Additionally, measures of bias, standard deviation of differences, Pearson’s correlation and intraclass correlation were analysed to explore overall agreement between the two devices. No significant differences in mean and variance between the two devices were detected. Pearson’s correlation coefficients of averaged gait estimates were higher than 0.98 and 0.8, respectively, for unipedal and bipedal gait parameters, supporting a high level of agreement between the two devices. The connected insoles are therefore a device equivalent to GAITRite^®^ to estimate the mean and variability of gait parameters.

## 1. Introduction

A large number of conditions, including physiological aging, can influence gait patterns. Disturbance of gait can be an important disease symptom and is evaluated by performing qualitative gait assessments, such as visual observations as well as by using specific clinical scales (e.g., Expanded Disability Status Scale for multiple sclerosis, the Unified Parkinson’s Disease Rating Scale for Parkinson’s disease (PD)) [1,2]. However, these and other descriptive routine clinical assessments are often not accurate enough to measure gait precisely or monitor changes sensitively over time.

Understanding gait patterns and related gait parameters and being able to characterise gait changes over time has an enormous potential for the general understanding of mobility [3]. It can serve as a predictor of morbidity and mortality [4], can lead to an early differential diagnosis [5,6], can allow the monitoring of efficacy of drug therapies [7] or progression of rehabilitation [8], can be used in the assessment of risk of falls [9] and can be evaluated in several other as yet unexploited indications. Gait variability, which is defined as the percent range of variance describing the regularity and consistency of a step cycle, is directly linked to dynamic postural control [10,11]. In a healthy subject, the gait parameters can vary naturally by around 7% between each step [12]. A higher variability in terms of magnitude and dynamics often reflects an impairment of gait, which is typically observed in movement disorders such as PD or Huntington’s disease [10,13]. Several studies demonstrated that a high variability of gait can result in walking instability and a higher risk of falling [14]. The estimation of this variability of gait requires a tool that can provide a large amount of robust and accurate quantifiable data, and one that is easy to use.

There are several devices available to measure gait parameters, each having their own limitations with respect to the above.

Laboratory gold-standard methods such as GAITRite^®^ (CIR Systems, Inc., Sparta, NJ, USA) or force-plates with optoelectronic motion capture systems are highly accurate, using ground contact force calculated using a matrix of pressure sensors in the case of GAITRite^®^. However, their use is time- and space-consuming, they cannot be applied outside gait analysis laboratory settings and the amount of data generated is limited [15,16].

Regarding the different solutions using the Inertial Measurement Unit (IMU), those directly attached to the foot provided the best performance compared to wearables positioned on other parts of the body [17,18]. This technology uses acceleration and angular velocity from the IMU to detect heel strike (HS) and toe-off (TO). However, as HS and TO events are defined by the presence or absence of a ground reaction force or plantar pressure [12], the use of pressure parameters is theoretically more relevant than acceleration and angular velocity to detect these events accurately. This is supported by the fact that the majority of devices with IMU were compared to pressure sensors for the validation of temporal gait events [19].

**Figure A1:**
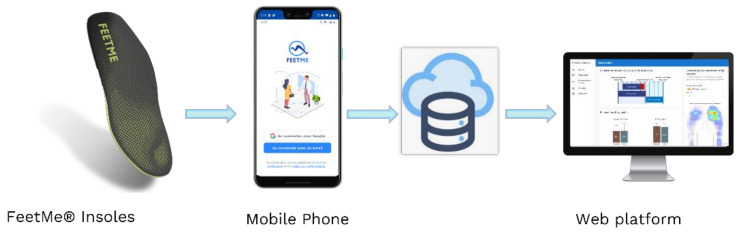
FeetMe^®^ Monitor solution includes the insoles, and the mobile application to collect data and to transfer to the web platform. The web platform shows the gait parameters and the pressure measurements.

Several studies have proposed prototypes that combine pressure sensors with an inertial measurement system to assess temporal gait events. However, these prototypes were either poorly optimized for everyday use [20,21], or require the use of a specific orthosis [22]. Recently, there has been an emergence of commercially available solutions combining inertial and pressure sensors in response to the need for ergonomics wearable for everyday life [20]. The FeetMe^®^ insoles (Figure A1 of the Appendix A) integrate both the derived ground reaction force by the built-in 19 pressure sensors to detect HS and TO, and the acceleration and angular velocity from the IMU to estimate spatial gait parameters. The estimation of stride parameters is embedded in each insole, while the time synchronization of the pair of insoles is carried out with a mobile phone. Unlike other insoles, these insoles enable the embedded estimation of gait temporal events and stride length, and the gait data are stored within the insoles themselves. No data transfer is therefore required for the calculation of these parameters. This also allows the insoles to be used as a standalone device without any connection to a smartphone, enabling fully passive remote monitoring in real-world settings for a duration of up to 13 days.

The current experiment has been designed as a validation study to evaluate the accuracy and reliability of gait metrics measurements by the FeetMe^®^ Monitor. Here, the FeetMe^®^ Monitor is compared to an established laboratory assessment tool, the GAITRite^®^ mat, in healthy volunteers.

## 2. Materials and Methods

### 2.1. Device and Algorithms

An insole (FeetMe^®^ Monitor, Paris, France) includes one IMU with the following specifications: accelerometer range, 8 g; gyroscope range, 1000 dps; and sampling frequency, 140 Hz, and nineteen capacitive cells with the following specifications: sampling frequency, 110 Hz; 8 bit digital signal; and 15 mm² cell area. The GAITRite® Platinum mat has a dimension of 0.61 × 7.92 m² and a sampling frequency of 60 Hz.

In the insoles, HS and TO were detected by the capacitive cell pressure sensors and the related temporal parameters such as swing, stance and step, and single support durations were calculated from HS and TO event timings. HS detection is based on two signals: the sum of the sensor signal of each cell (S) and the derivative of the sum of sensor signals (dS/dt). The calculation of the derivative and the sum of sensor signals were filtered by the Savitzky–Golay filter. The condition of HS was:

If dS/dt > 0.2 at time(i) and if at time(i) + 100ms S > 50 then HS is detected at time (i).
HS is not updated until the next TO detection.

The condition of TO detection was based on one signal, a threshold of S, and was:

If S < 30 at time(i) and if at time(i) + 100ms S < 30 then TO is detected at time (i).
TO is not updated until the next HS detection.

**Figure 1:**
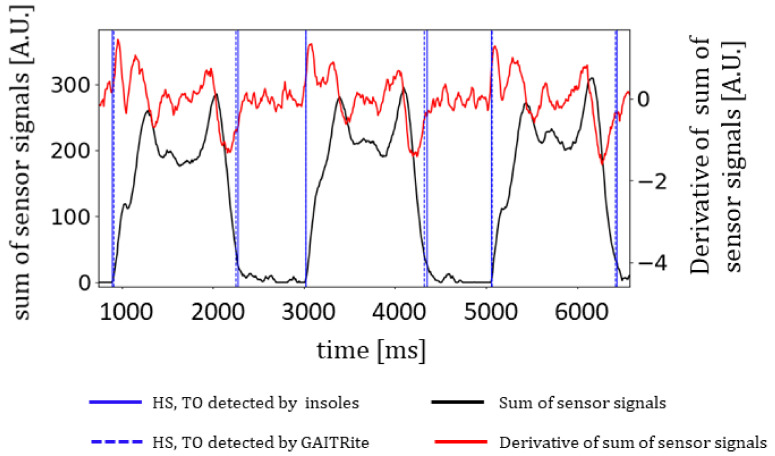
Detection of HS and TO events by the insoles and GAITRite^®.^

Figure 1 illustrates the sum of sensor signals (black line) and its derivative (red line) in arbitrary units (A.U.). HS and TO events as detected by the insoles and GAITRite^®^ are depicted in blue solid and dashed lines, respectively. To synchronize insoles and GAITRite^®^ records, the first event of HS of insoles and GAITRite^®^ of each run was forced to be equal to zero by subtracting the value from subsequent HS and TO events for each device. This first measure was then removed from the dataset.

Stride length was calculated by integrating the acceleration two times and by removing drift values with a Kalman filter [17]. HS, TO and stride length were calculated in each insole and are defined as *insole-based* parameters. For each insole, these parameters were sent to a mobile phone by Bluetooth Low Energy (BLE). The velocity, stance, swing, step and single support durations and cadence defined as *mobile-based* parameters were calculated by a dedicated mobile application from the insole-based parameters.

### 2.2. Study Design and Procedures

The study included 30 healthy volunteers to compare gait parameters measured by 2 devices used concurrently: GAITRite^®^ and insoles. Each subject was evaluated simultaneously on the GAITRite^®^ mat and the FeetMe^®^ Monitor insoles (FeetMe^®^ S.A.S., Paris, France). Each subject completed three 8-metre trials of gait at a comfortable pace. These measures were repeated 4 times per day on 2 days separated by a week. The participants kept the same shoes at both sessions. The insoles were placed in the subject’s shoes and calibrated by the raters. The calibration procedure consisted of putting the insoles in the patient’s shoes and resetting all the sensor values to zero by clicking a button in the FeetMe^®^ Monitor mobile application. Then, the patients put on their shoes and were asked to stand in a defined area in front of the mat with both feet together. As they started walking onto the mat, the rater started the recording. The subjects stopped with feet together right after the mat while the rater stopped recording on FeetMe^®^ insoles. Likewise, the clinician started and saved the record on GAITRite^®^ software before and after the walk, respectively. All the raters applied the same procedure.

The study was conducted in the Neurorehabilitation Department at University Hospital Henri Mondor in Créteil, France. The study was performed in accordance with the Declaration of Helsinki (2008), Good Clinical Practice (GCP) guidelines and local regulatory requirements (registration number, ID-RCB: 2018-A01388-47). Each subject provided written informed consent prior to their participation in the study.

### 2.3. Statistical Analysis

As a first step, the accuracy of insole-based parameters including HS, TO and stride length was characterized by Bland–Altman plots, the bias, the SD of differences, the 95th confidence interval (CI) of differences and the mean absolute of differences between GAITRite^®^ and insoles.

The gait parameters analysis was divided into two categories: the unipedal gait parameters (stride length, velocity, stance and swing durations and cadence), and the bipedal parameters (step and single support durations). The average estimates and the estimates of variability were calculated for each subject. The average estimate is the average value of all steps per subject. The estimate of variability is the coefficient of variation of all steps of each subject and is expressed as a percentage (%). Means, standard deviations and the corresponding confidence intervals of each gait parameter estimated by GAITRite^®^ and insoles were calculated. A *t*-test from two independent samples and a Levene’s test were also calculated for each estimate to detect any significant differences between GAITRite^®^ and insoles. There was no adjustment for multiple comparisons.

Bias, SD of the differences, Pearson’s correlation coefficient and the intra class correlation coefficient (ICC (2,1): two-way random with absolute agreement) were calculated for each estimate (i.e., mean and variability) of gait parameters. The bias measurement between the insoles and GAITRite^®^ represents the systematic error. The SD of the differences between the insoles and GAITRite^®^ represents the random error. The linear regressions were applied to quantify the similarity of measurement between mat and insoles.

All statistical calculations were performed in Python 3.8 and RStudio 1.4.

## 3. Results

### 3.1. Demographics

A total of 30 healthy volunteers, 12 females and 18 males, were included in the study. Participants ranged in age from 20 to 77 years with a mean of 47. Average height and weight were 172 ± 11 cm and 76 ± 17 kg, respectively. Out of the 30 subjects assessed, 5 were excluded from the analysis due to incomplete data capture (manipulation errors, crash of the mobile application and loss of Bluetooth Low Energy connection). The 25 remaining subjects generated altogether 4830 HS and TO events.

### 3.2. Insole-Based Parameters

Table 1 summarizes the statistics of differences in the insole-based parameters estimated at each step compared to GAITRite^®^ with the following statistics: biases, SD of differences, 95th CI of differences and the mean absolute differences.

**Figure 2:**
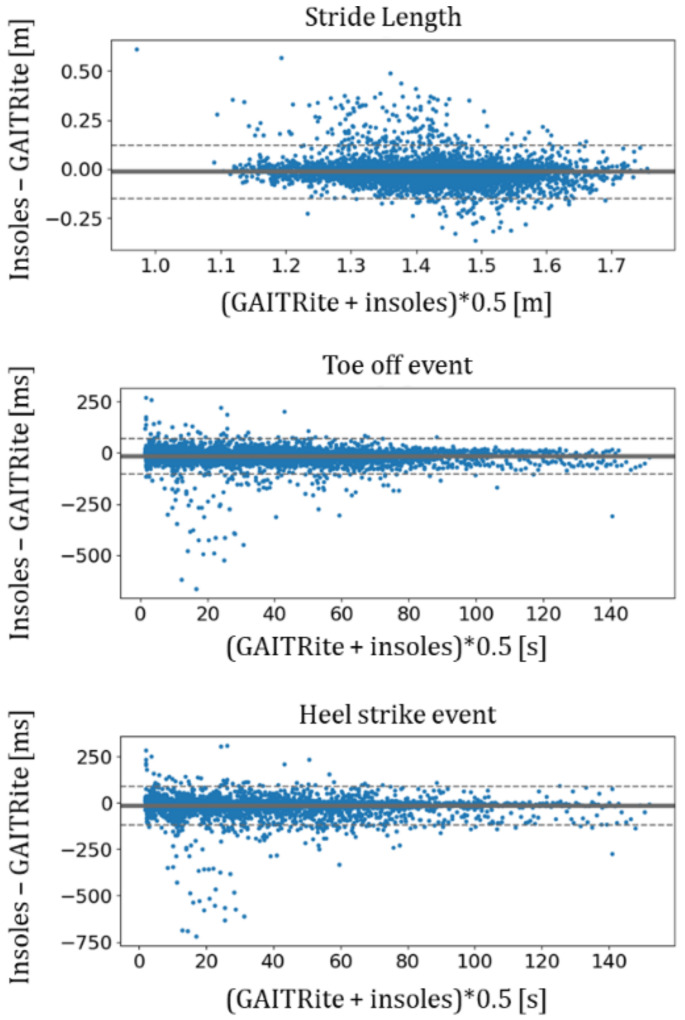
Bland–Altman plots of insole-based parameters including HS, TO and stride length. The solid line is the bias, and the dashed lines are the lower and upper limits of the 95th CI of differences.

For these three insole-based parameters, the biases were very low, with 17 ms for HS and TO and 1.4 cm for stride length. Indeed, these values were close to zero, as shown on the Bland–Altman plots in Figure 2. The SDs of the differences were more than twice as high as the biases.

### 3.3. Analysis of Average Estimates of Unipedal Gait Parameters: Velocity, Stride Length, Stance and Swing Durations and Cadence

**Figure 3:**
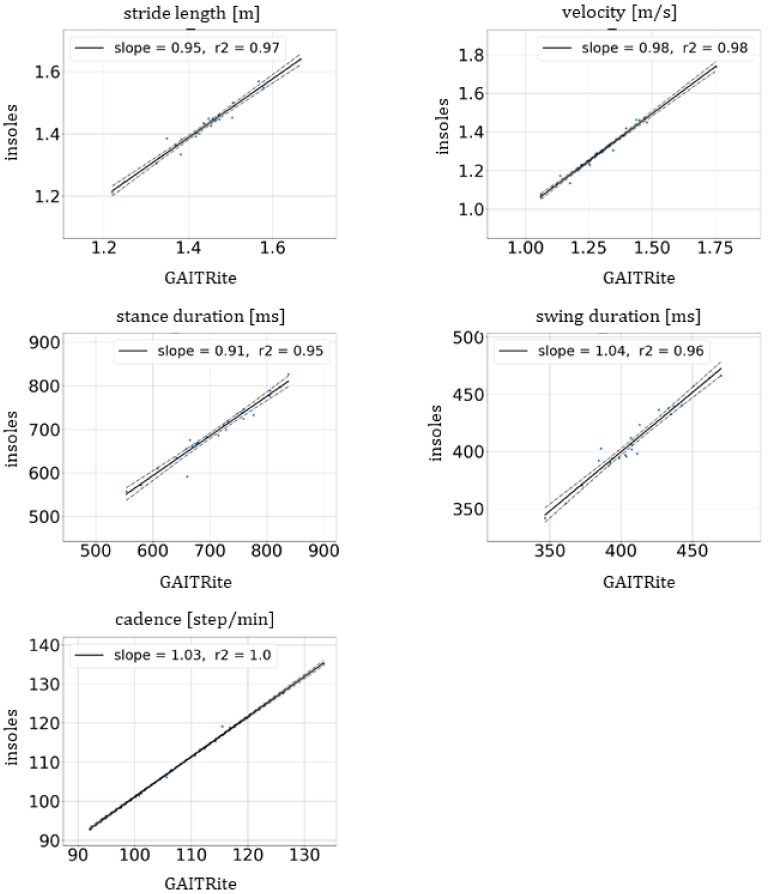
Linear regression of average estimates of unipedal gait parameters for velocity, stride length, stance and swing duration and cadence between GAITRite^®^ mat and the insoles (solid lines). The dashed lines represent the confidence region. The values of the slope and the coefficient of determination, r², are provided in the legends.

For the mean and SD provided with CI in Table 2, the estimates were very close between the insoles and GAITRite^®^. No significant difference was observed between averages of GAITRite^®^ and insoles and every *p*-value was greater than 0.4, while the threshold significance level was 0.05. Similarly, no significant difference in SD was observed with Levene’s test between the two devices. Figure 3 shows the linear regression of the average estimates for all patients between mat and insoles for each unipedal gait parameter. All slopes and the coefficient of determination, r², were, respectively, between 0.91 and 1.04 and between 0.95 and 1.0. The Pearson’s correlation and ICC (2,1) values which were, respectively, greater than or equal to 0.98 and 0.95 reinforce the good result of linear regressions as shown in Table 3. The correlation values are therefore defined as excellent.

### 3.4. Estimates of Variability of Unipedal Gait Parameters

**Figure 4:**
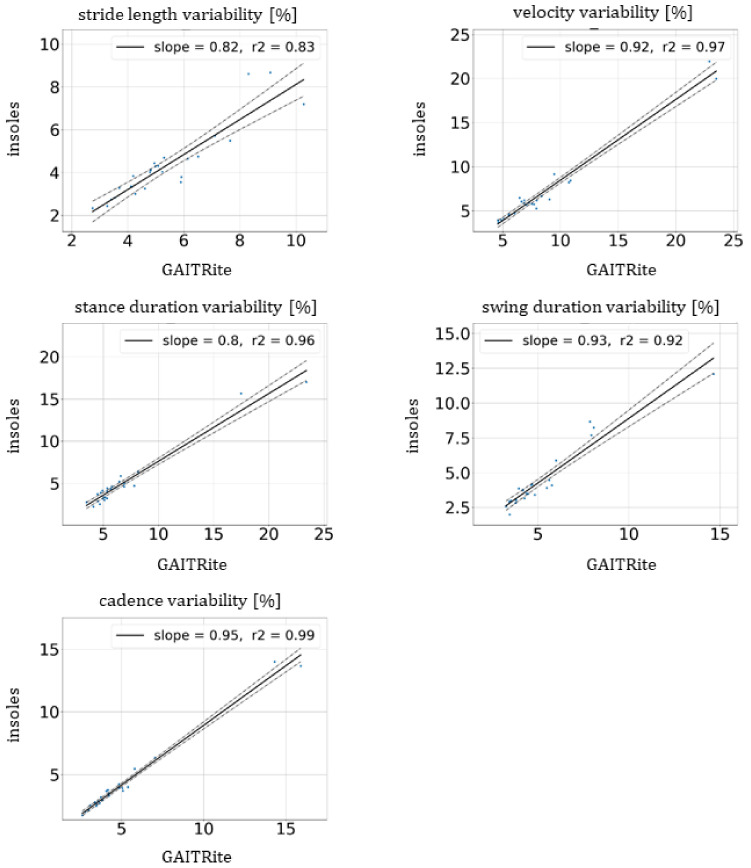
Linear regression of estimates of variability (coefficient of variation) of unipedal gait parameters for velocity, stride length, stance and swing duration and cadence between GAITRite^®^ mat and the insoles (solid lines). The dashed lines represent the confidence region. The values of the slope and the coefficient of determination, r², are provided in the legends.

No significant difference in means and SD were observed between the two devices as all *p*-values were greater than 0.06 (at a significance level of 0.05) with *t*-test and Levene’s tests. For the mean and SD, the estimates of variability were very close between the insoles and GAITRite^®^, as shown in Table 3. Figure 4 illustrates the linear regression of the estimates of variability for each patient between mat and insoles for all unipedal gait parameters. All slopes and r² were, respectively, between 0.8 and 0.95 and between 0.83 and 0.99.

The Pearson’s correlation coefficients were greater than or equal to 0.91, which highlights excellent correlations. For velocity, stance and swing durations and cadence, the ICC (2,1) values were higher than or equal to 0.88, which reinforces this excellent agreement. The ICC (2,1) of the stride length was equal to 0.77 (see Table 3).

The measures of differences such as bias and SD of differences are provided in Table 3. The biases of variability were lower than 2% for all unipedal gait parameters to guarantee an accurate estimation.

### 3.5. Analysis of Average Estimates of Bipedal Gait Parameters: Step Duration and Single Support Duration

**Figure 5:**
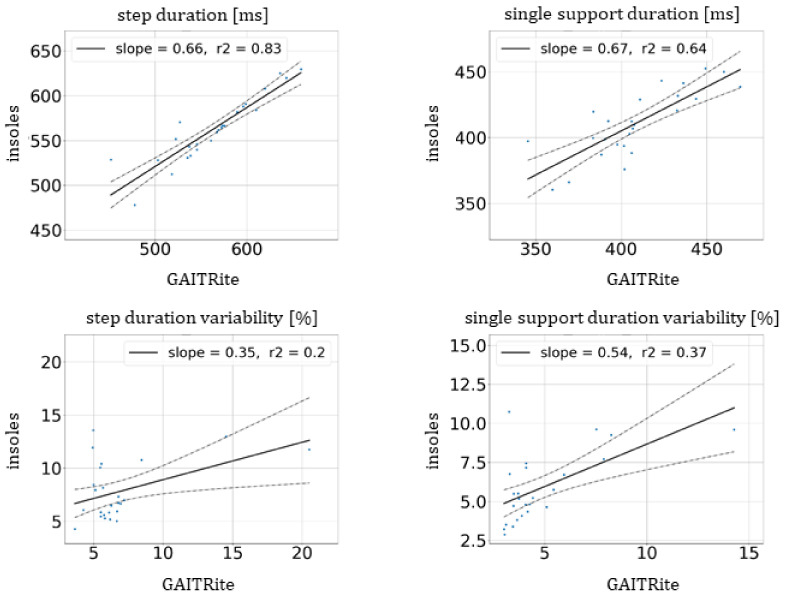
Linear regression of average estimates and estimates of variability of bipedal gait parameters with step duration and single support duration between GAITRite^®^ mat and the insoles (solid lines). The dashed lines represent the confidence region. The values of the slope and the coefficient of determination, r², are provided in the legends.

No significant differences in the average estimates between GAITRite^®^ and the insoles were observed, and all *p*-values of *t*-tests and Levene’s tests were higher than 0.15 (at a significance level of 0.05) as shown in Table 4. Indeed, regarding the mean and SD measured, the average estimates were very close between the insoles and GAITRite^®^. Figure 5 illustrates the linear regression of the average estimates between the mat and insoles for bipedal gait parameters. The slopes were, respectively, equal to 0.66 and 0.67 for step and single support durations. Moreover, the Pearson correlation and ICC (2,1) values of step duration given in Table 5 were excellent and equal, respectively, to 0.91 and 0.87. For the single support duration, the values were good and, respectively, equal to 0.8 and 0.79 [23,24].

### 3.6. Estimates of Variability of Bipedal Gait Parameters: Step Duration and Single Support Duration

As shown in Table 4, no significant differences in the estimates of variability of the step and single support duration between GAITRite^®^ and insoles were observed, with *p*-values of 0.26 and 0.13, respectively (at a significance level of 0.05). The regression slopes were lower than for the average estimates with 0.35 and 0.54, respectively, for step and single support durations (Figure 5).

The bias ± SD of differences in the step and single support duration were higher than 2% and were, respectively, 0.977 ± 3.312% and 1.026 ± 2.073%. Although these values may seem very low, the Pearson’s correlation and ICC (2,1) values for the variability of bipedal metrics were much lower than in case of the unipedal parameters. For the variability of the single support duration, they were equal to 0.61 and 0.56, respectively, and for the variability of the step duration, all the values were lower than 0.5.

## 4. Discussion

### 4.1. Insole-Based Parameters

Panebianco et al., (2018) compared 17 algorithms, estimating HS and TO by taking into account the accuracy and the location of the IMU [18]. In their study, an IMU directly attached to the feet provided the best performance compared to wearables positioned on the trunk or the ankle on healthy volunteers. The median errors for detecting HS or TO ranged from 60 to 65 ms when compared to GAITRite^®^. In our study, mean absolute differences in HS and TO were equal to 28 and 27 ms, respectively. These latter values demonstrate the better performance of these FeetMe^®^ insoles to detect HS and TO events compared to IMU alone.

We found that stride length had a standard deviation of differences equal to 6.9 cm, and this value was equivalent to those found by Ferrari et al. [25]. In their study, the standard deviations of differences between IMU and the video camera were close to 7 cm. The calculations of HS and TO embedded in the insole have better performance than in the study of Panebianco et al., for the stride length, the same performance as observed by Ferrari et al. Unlike Ferrari et al., where the calculation was carried out by the mobile phone, in our study the calculation of stride length was carried out in the insole (without using the phone). Such embedded calculation allows the use of the insoles without a mobile phone and to have a standalone wearable for several days.

### 4.2. Mean and Variability Estimates of Gait Parameters

For the stride length, stance and swing durations, the bias ± SD of differences between insoles and GAITRite^®^ were, respectively, −1.3 ± 1.6 cm, −16.3 ± 16.5 ms and −0.011 ± 6.76 ms. With a spatial and temporal resolution of 1.27 cm and 16.7 ms of the GAITRite^®^ mat, the bias and SD of differences in FeetMe^®^ insoles were equal to or lower than the precision of the latter. When the differences (bias and SD) between the insoles and the GAITRite^®^ mat are close to the spatial and temporal resolution of GAITRite^®^, the accuracy of both devices becomes equivalent.

A first insight into this high temporal accuracy is the results of variability estimates. For all unipedal temporal parameters, either the mean or variability estimates, the correlations were excellent (superior to 0.90). A second insight into this high spatial and temporal accuracy is the excellent correlations (equal to 0.99) between the mean and variability estimates for the velocity.

The Pearson’s correlations and ICC(2,1) values reported for average estimates were excellent for all gait parameters, except for the single support duration, which was good [23,24]. These excellent correlations were in the same range as the values reported in the validation paper of GAITRite^®^ mat [15]. The Pearson’s correlations and ICC (2,1) of the variability of the unipedal gait parameters were excellent, except for the ICC (2,1) of the stride length, which was good. For the variability estimates of the bipedal parameters, the Pearson’s correlation and ICC (2,1) of the single support duration were, respectively, moderate and fair. All correlations in step duration variability were poor between GAITRite^®^ and insoles. The lower accuracy in the variability of step duration could be explained by the synchronization between the left and right insoles. Indeed, the reference time of the two insoles can be slightly different because of the BLE transmission from the insole to the mobile phone. An improved synchronization between the timestamps of the two insoles would lead to an improvement in the precision of the variability of the step duration.

### 4.3. Clinical Applicability

The validation of mean and variability estimates opens many new perspectives for both controlled standard clinical tests and real-life data collection.

For example, the output of the 6-min walking test (6-MWT) which delivers the distance cannot be estimated on a short GAITRite^®^ mat [26]. The insoles enable the gait assessment at each step and the total distance can be easily calculated by the sum of stride lengths. Since the insoles do not require a laboratory set up and are not restricted in space (as the GAITRite^®^ mat or optoelectronic cameras are), the 6-MWT and other standard gait assessments become readily accessible to all healthcare professionals and even directly to patients without loss of accuracy.

Nevertheless, many conditions need assessment beyond the standard tests and require the collection of data throughout a long period of time in the real world [27]. A first real-life study with FeetMe^®^ insoles showed that walking while talking results in increased gait cycle and step time, with a prolonged stance phase. Variability of gait cycle and stance phase also increased during the most demanding dual task [28]. These results support the theory of the existing association between cognition and gait, as highlighted by Byun et al. [29]. They showed that gait variability can predict the risk of cognitive decline in cognitively normal elderly people.

Gait variability might also be a gait feature to distinguish the Parkinsonian form of multiple system atrophy (MSA-P) from Parkinson’s disease (PD). Significant differences of 4% in the variability of stride length and velocity between PD and MSA-P patients were demonstrated in the study by Sidoroff et al. [30]. The insoles could measure these parameters and support the otherwise challenging differential diagnosis of these patients.

Fall risk assessment is a major public health interest. To identify subjects at risk, wearable devices to assess gait patterns indicative of the risk to fall could be deployed. Verghese et al. showed that a decrease in walking speed of 10cm/s increases the risk ratio for falls [9]. With [−4, 4] cm/s corresponding to the 95th CI of differences, the insoles tested in the current study accurately detect a clinically meaningful difference in velocity for the assessment of the risk ratio.

### 4.4. Population Assessed

This study validated the gait parameters in healthy volunteers. Previously, the FeetMe^®^ insoles had been validated against GAITRite^®^ in two non-healthy groups with gait disorders, i.e., in stroke and multiple sclerosis patients [31,32]. These studies suggest that the mean estimates and variability measures are also accurate in these and possibly other populations.

## 5. Conclusions

Based on all the gait parameters measured, including stride length, velocity, stance, swing, step and single support durations and cadence, the average estimates between the insoles and GAITRite^®^ mat data did not show a statistically significant difference (at the 0.05 level). The bias or the SD of differences in the insoles were close to the spatial or temporal resolution of the mat, with −1.3 ± 1.6 cm, −16.3 ± 16.5 ms and −0.011 ± 6.76 ms for the stride length, the stance and swing durations, respectively. For the unipedal parameters, the estimates of variability with the insoles were accurate, with a Pearson’s correlation higher than 0.91. For clinical gait assessment in multiple pathologies, these insoles are promising, with the accuracy and the reliability of a laboratory assessment tool, but with the additional capability of collecting data continuously over long periods of time and in real-world settings.

## Figures and Tables

**Table 1:** Characterization of the differences between GAITRite^®^ and insoles for the insole-based parameters including HS, TO events and stride length with 4830 samples.

Insole-Based Parameters	N	Bias	SD of Differences	Lower Limit of 95th CI	Upper Limit of 95th CI	Mean Absolute Differences
Heel Strike (ms)	4830	−17	53	−121	87	28
Toe Off (ms)	4830	−17	44	−104	69	27
Stride Length (m)	4830	−0.014	0.069	−0.15	0.12	0.046

**Table 2:** Values of average estimates and estimates of variability for the unipedal gait parameters with the following statistics: sample size (N), mean and standard deviations (SD) with GAITRite^®^, mean and standard deviations (SD) with insoles and p-values of *t*-test with independent samples and Levene’s test between GAITRite^®^ and the insoles.

	N	Mean GAITRite^®^ Lower Limit [Mean] Upper Limit	Mean Insoles Lower Limit [Mean] Upper Limit	SD GAITRite^®^ Lower Limit [Mean] Upper Limit	SD Insoles Lower Limit [Mean] Upper Limit	*t*-Test *p*-Value	Levene’s Test *p*-Value
Average Estimates							
Stride Length (m)	25	1.388 [1.43] 1.471	1.375 [1.415] 1.455	0.078 [0.1] 0.139	0.075 [0.096] 0.134	0.64	0.84
Stride Velocity (m/s)	25	1.24 [1.305] 1.37	1.232 [1.295] 1.358	0.123 [0.157] 0.218	0.119 [0.153] 0.213	0.96	0.85
Stance Duration (ms)	25	670.5 [700.5] 730.4	661.1 [688.7] 716.4	56.6 [72.5] 100.9	52.3 [67.0] 93.2	0.42	0.64
Swing Duration (ms)	25	397.0 [409.1] 421.2	396.5 [409.4] 422.3	22.9 [29.3] 40.8	24.5 [31.4] 43.6	0.85	0.72
Cadence (step/min)	25	105.3 [109.3] 113.4	106.0 [110.2] 114.3	7.7 [9.8] 13.7	7.8 [10.0] 13.9	0.65	0.88
**Estimates of Variability**							
Stride Length Variability (%)	25	4.724 [5.506] 6.288	3.671 [4.361] 5.052	1.479 [1.894] 2.635	1.306 [1.673] 2.328	0.06	0.77
Stride Velocity Variability (%)	25	5.915 [7.957] 9.998	5.032 [6.88] 8.729	3.862 [4.946] 6.881	3.497 [4.479] 6.231	0.29	0.94
Stance Duration Variability (%)	25	3.5 [5.432] 7.364	3.059 [4.554] 6.05	3.655 [4.681] 6.511	2.83 [3.624] 5.041	0.13	0.88
Swing Duration Variability (%)	25	4.154 [5.139] 6.124	3.449 [4.403] 5.356	1.863 [2.386] 3.32	1.804 [2.31] 3.214	0.26	0.91
Cadence Variability (%)	25	2.955 [4.362] 5.77	2.708 [3.995] 5.281	2.663 [3.41] 4.744	2.433 [3.117] 4.336	0.35	0.93

**Table 3:** Values of average estimates and estimates of variability for the unipedal gait parameters with the following statistics: sample size (N), the bias: mean of (insoles-GAITRite^®^), SD of differences: SD of (insoles-GAITRite^®^), lower and upper limits of 95th CI of differences, Pearson’s correlation and intra-class correlation coefficients between GAITRite^®^ and the insoles. ^(^*^)^ Note that the CI for the CV estimates are approximate only, based on t-intervals, as the underlying distribution is not known.

	N	Bias	SD of Differences	Lower Limit of 95th CI of Differences	Upper Limit of 95th CI of Differences	Pearson’s Correlation	ICC (2,1)
Average Estimates							
Stride Length (m)	25	−0.013	0.016	−0.05	0.02	0.99	0.98
Stride Velocity (m/s)	25	−0.002	0.019	−0.04	0.04	0.99	0.99
Stance Duration (ms)	25	−16.336	16.503	−48.68	16.01	0.98	0.95
Swing Duration (ms)	25	−0.011	6.761	−13.26	13.24	0.98	0.98
Cadence (step/min)	25	1.282	0.602	0.1	2.46	1	0.99
**Estimates of Variability**							
Stride Length Variability (%)	25	−1.061	0.764	−2.56 ^(^*^)^	0.44 ^(^*^)^	0.91	0.77
Stride Velocity Variability (%)	25	−1.404	0.852	−3.07 ^(^*^)^	0.27 ^(^*^)^	0.99	0.94
Stance Duration Variability (%)	25	−1.751	1.123	−3.95 ^(^*^)^	0.45 ^(^*^)^	0.98	0.88
Swing Duration Variability (%)	25	−0.768	0.68	−2.1 ^(^*^)^	0.57 ^(^*^)^	0.96	0.91
Cadence Variability (%)	25	−0.837	0.406	−1.63 ^(^*^)^	-0.04 ^(^*^)^	0.99	0.96

**Table 4:** Values of average estimates and estimates of variability for the bipedal gait parameters with the following statistics: sample size (N), mean and standard deviations (SD) with GAITRite^®^, mean and standard deviations (SD) with insoles and *p*-values of *t*-test with independent samples and Levene’s test between GAITRite^®^ and the insoles.

	N	Mean GAITRite^®^ Lower Limit [Mean] Upper Limit	Mean Insoles Lower Limit [Mean] Upper Limit	SD GAITRite^®^ Lower Limit [Mean] Upper Limit	SD Insoles Lower Limit [Mean] Upper Limit	*t*-Test *p*–Value	Levene’s Test *p*–Value
**Average Estimates**							
Step Duration (ms)	25	542 [563] 584	547 [562] 577	39.2 [50.3] 69.9	28.3 [36.3] 50.5	0.93	0.15
Single Support Duration (ms)	25	396 [408] 421	400 [410] 421	23.5 [30.1] 41.9	19.7 [25.2] 35.0	0.76	0.68
**Estimates of** **Variability**							
Step Duration Variability (%)	25	5.37 [6.81] 8.25	6.67 [7.79] 8.91	2.72 [3.48] 4.85	2.12 [2.72] 3.78	0.27	0.56
Single Support Duration Variability (%)	25	3.81 [4.83] 5.85	4.94 [5.85] 6.76	1.93 [2.47] 3.43	1.72 [2.20] 3.06	0.13	0.54

**Table 5:** Values of average estimates and estimates of variability for the bipedal gait parameters with the following statistics: sample size (N), the bias: mean of (insoles-GAITRite^®^), SD of differences: SD of (insoles-GAITRite^®^), lower and upper limits of 95th CI of differences, Pearson’s correlation and intra-class correlation coefficients between GAITRite^®^ and the insoles. ^(^*^)^ Note that the CI for the CV estimates are approximate only, based on t-intervals, as the underlying distribution is not known.

	N	Bias	SD of Differences	Lower Limit of 95th CI of Differences	Upper Limit of 95th CI of Differences	Pearson’s Correlation	ICC (2,1)
**Average Estimates**							
Step Duration (ms)	25	−0.52	22.647	−44.91	43.87	0.91	0.87
Single Support Duration (ms)	25	2.386	18.086	−33.06	37.83	0.8	0.79
**Estimates of Variability**							
Step Duration Variability (%)	25	0.977	3.312	−5.52 ^(^*^)^	7.47 ^(^*^)^	0.45	0.43
Single Support Duration Variability (%)	25	1.026	2.073	−3.04 ^(^*^)^	5.09 ^(^*^)^	0.61	0.56

## Data Availability

The data are available in a publicly accessible repository. The data presented in this study are openly available in [repository name, e.g., FigShare] at [doi], reference number [reference number].

## References

[1] Kurtzke J.F. (1983). Rating neurologic impairment in multiple sclerosis: An expanded disability status scale (EDSS). Neurology.

[2] Goetz C.G., Tilley B.C., Shaftman S.R., Stebbins G.T., Fahn S., Martinez-Martin P., Poewe W., Sampaio C., Stern M.B., Dodel R. (2008). Movement Disorder Society-sponsored revision of the Unified Parkinson’s Disease Rating Scale (MDS-UPDRS): Scale presentation and clinimetric testing results. Mov. Disord..

[3] Hnatiuc M., Geman O., Avram A., Gupta D., Shankar K. (2021). Human Signature Identification Using IoT Technology and Gait Recognition. Electronics.

[4] White D.K., Neogi T., Nevitt M.C., Peloquin C.E., Zhu Y., Boudreau R., Cauley J.A., Ferrucci L., Harris T.B., Satterfield S.M. (2012). Trajectories of Gait Speed Predict Mortality in Well-Functioning Older Adults: The Health, Aging and Body Composition Study. J. Gerontol. Ser. A Boil. Sci. Med. Sci..

[5] Agurto C., Heisig S., Abrami A., Ho B.K., Caggiano V. (2021). Parkinson’s disease medication state and severity assessment based on coordination during walking. PLoS ONE.

[6] Abrami A., Heisig S., Ramos V., Thomas K.C., Ho B.K., Caggiano V. (2020). Using an unbiased symbolic movement representation to characterize Parkinson’s disease states. Sci. Rep..

[7] Filli L., Zörner B., Kapitza S., Reuter K., Lörincz L., Weller D., Sutter T., Killeen T., Gruber P., Petersen J.A. (2017). Monitoring long-term efficacy of fampridine in gait-impaired patients with multiple sclerosis. Neurology.

[8] Baker R. (2006). Gait analysis methods in rehabilitation. J. Neuroeng. Rehabil..

[9] Verghese J., Holtzer R., Lipton R.B., Wang C. (2009). Quantitative Gait Markers and Incident Fall Risk in Older Adults. J. Gerontol. Ser. A Boil. Sci. Med. Sci..

[10] Hausdorff J.M., Cudkowicz M.E., Firtion R., Wei J.Y., Goldberger A.L. (1998). Gait variability and basal ganglia disorders: Stride-to-stride variations of gait cycle timing in parkinson’s disease and Huntington’s disease. Mov. Disord..

[11] Hausdorff J.M., Rios D.A., Edelberg H.K. (2001). Gait variability and fall risk in community-living older adults: A 1-year prospective study. Arch. Phys. Med. Rehabil..

[12] Perry J., Burnfield J.M. (2010). Gait Analysis: Normal and Pathological Function.

[13] Hausdorff J.M. (2005). Gait variability: Methods, modeling and meaning. J. Neuroeng. Rehabil..

[14] Brach J.S., Perera S., Studenski S., Katz M., Hall C., Verghese J. (2010). Meaningful change in measures of gait variability in older adults. Gait Posture.

[15] Webster K.E., Wittwer J., Feller J.A. (2005). Validity of the GAITRite® walkway system for the measurement of averaged and individual step parameters of gait. Gait Posture.

[16] Windolf M., Götzen N., Morlock M. (2008). Systematic accuracy and precision analysis of video motion capturing systems—exemplified on the Vicon-460 system. J. Biomech..

[17] Trojaniello D., Ravaschio A., Hausdorff J.M., Cereatti A. (2015). Comparative assessment of different methods for the estimation of gait temporal parameters using a single inertial sensor: Application to elderly, post-stroke, Parkinson’s disease and Huntington’s disease subjects. Gait Posture.

[18] Panebianco G.P., Bisi M.C., Stagni R., Fantozzi S. (2018). Analysis of the performance of 17 algorithms from a systematic review: Influence of sensor position, analysed variable and computational approach in gait timing estimation from IMU measurements. Gait Posture.

[19] Prasanth H., Caban M., Keller U., Courtine G., Ijspeert A., Vallery H., von Zitzewitz J. (2021). Wearable Sensor-Based Real-Time Gait Detection: A Systematic Review. Sensors.

[20] Arafsha F., Hanna C., Aboualmagd A., Fraser S., El Saddik A. (2018). Instrumented Wireless SmartInsole System for Mobile Gait Analysis: A Validation Pilot Study with Tekscan Strideway. J. Sens. Actuator Netw..

[21] Chen W., Xu Y., Wang J., Zhang J. (2016). Kinematic Analysis of Human Gait Based on Wearable Sensor System for Gait Rehabilitation. J. Med. Biol. Eng..

[22] Kwon J., Park J.-H., Ku S., Jeong Y., Paik N.-J., Park Y.-L. (2019). A Soft Wearable Robotic Ankle-Foot-Orthosis for Post-Stroke Patients. IEEE Robot. Autom. Lett..

[23] Koo T.K., Li M.Y. (2016). A Guideline of Selecting and Reporting Intraclass Correlation Coefficients for Reliability Research. J. Chiropr. Med..

[24] Akoglu H. (2018). User’s guide to correlation coefficients. Turk. J. Emerg. Med..

[25] Ferrari A., Ginis P., Hardegger M., Casamassima F., Rocchi L., Chiari L. (2015). A Mobile Kalman-Filter Based Solution for the Real-Time Estimation of Spatio-Temporal Gait Parameters. IEEE Trans. Neural Syst. Rehabil. Eng..

[26] ATS Committee on Proficiency Standards for Clinical Pulmonary Function Laboratories (2002). ATS statement: Guidelines for the six-minute walk test. Am. J. Respir. Crit. Care Med..

[27] Morgan C., Rolinski M., McNaney R., Jones B., Rochester L., Maetzler W., Craddock I., Whone A.L. (2020). Systematic Review Looking at the Use of Technology to Measure Free-Living Symptom and Activity Outcomes in Parkinson’s Disease in the Home or a Home-like Environment. J. Park. Dis..

[28] Lunardini F., Malavolti M., Pedrocchi A.L.G., Borghese N.A., Ferrante S. (2021). A mobile app to transparently distinguish single- from dual-task walking for the ecological monitoring of age-related changes in daily-life gait. Gait Posture.

[29] Byun S., Han J.W., Kim T.H., Kim K., Park J.Y., Suh S.W., Seo J.Y., So Y., Lee K.H., Lee J.R. (2018). Gait Variability Can Predict the Risk of Cognitive Decline in Cognitively Normal Older People. Dement. Geriatr. Cogn. Disord..

[30] Sidoroff V., Raccagni C., Kaindlstorfer C., Eschlboeck S., Fanciulli A., Granata R., Eskofier B., Seppi K., Poewe W., Willeit J. (2020). Characterization of gait variability in multiple system atrophy and Parkinson’s disease. J. Neurol..

[31] Farid L., Jacobs D., Santos J.D., Simon O., Gracies J.-M., Hutin E. (2020). FeetMe® Monitor-connected insoles are a valid and reliable alternative for the evaluation of gait speed after stroke. Top. Stroke Rehabil..

[32] Domínguez A.G., Hochsprung A., Duarte S.P., Camino C.P., Rodríguez A.A., Durán C., Izquierdo G. (2020). Study for the Validation of the FeetMe® Integrated Sensor Insole System Compared to GAITRite® System to Assess the Characteristics of the Gait in Patients with Multiple Sclerosis (4038). Neurology.

